# Divergent virulence and serological profiles reveal pathogenic evolution within the *Bandavirus* genus

**DOI:** 10.1080/21505594.2026.2696699

**Published:** 2026-07-01

**Authors:** Chenxuan Li, Shu Shen, Liyan Fu, Jun Ni, Jian Xiao, Jin Qian, Shengyao Chen, Yaohui Fang, Min Zhou, Fei Deng, Xiaoli Wu

**Affiliations:** a Key Laboratory of Virology and Biosafety and National Virus Resource Center, Wuhan Institute of Virology, Chinese Academy of Sciences, Wuhan, China; b University of Chinese Academy of Sciences, Beijing, China; c Brain Science and Advanced Technology Institute, Wuhan University of Science and Technology, Wuhan, China; d Key Laboratory of Genetic Evolution & Animal Models, Yunnan International Joint Laboratory of Zoonotic Viruses, Yunnan Key Laboratory of Biodiversity Information, Kunming Institute of Zoology, Chinese Academy of Sciences, Kunming, Yunnan, China

**Keywords:** Tick-borne viruses, *Bandavirus*, pathogenicity differences, antigenic properties, cross-neutralization, viral evolution

## Abstract

Viruses within the *Bandavirus* genus (*Phenuiviridae* family), phylogenetically divided into SFTSV and Bhanja groups, pose public health risks through cross-species transmission. Despite their significance, a systematic comparison of their infectivity, pathogenicity, and antigenicity is lacking. This study evaluates these key properties for five representative species: Severe fever with thrombocytopenia syndrome virus (SFTSV), Guertu virus (GTV), and Heartland virus (HRTV) from the SFTSV group; and Bhanja virus (BHAV) and Lone Star virus (LSV) from the Bhanja group. The five bandaviruses demonstrated efficient replication kinetics across a panel of human and mammalian cell lines, suggesting a broad cellular tropism. Wild-type C57BL/6 mice developed transient viremia (2–10 dpi) without overt disease, followed by neutralizing antibody production. Cross-neutralization was robust within each phylogenetic group but restricted between groups, supporting two separate serogroups. In contrast, IFNAR^−/−^ C57BL/6 mice exhibited significant hematological abnormalities and uniformly lethal infection, with the 50% lethal dose–based virulence ranking as SFTSV > LSV > GTV >BHAV >HRTV. Pathological analysis revealed that SFTSV caused severe multi-organ damage, with predominant spleen tropism; GTV and HRTV induced similar but milder spleen-tropic lesions; and LSV and BHAV caused multi-organ injuries primarily affecting the liver and spleen. Our findings demonstrate that the variation in virulence among *Bandavirus* species is closely linked to their antigenic divergence and evolutionary lineages. The established infection models provide valuable platforms for investigating virus-host interactions and pathogenesis. These insights facilitate the development of vaccines and antiviral therapies, and improve public health preparedness against emerging tick-borne pathogens.

## SUMMARY

We studied a group of tick-borne viruses known as*Bandavirus*, which pose a growing threat to both human and animal health. These viruses can infect different hosts, cause disease, and vary in how the immune system recognizes them, yet they have not been carefully compared in a single study. To close this gap, we examined five types of*Bandavirus*using both cell cultures and mouse models. We found that all five viruses infect a wide range of mammalian cells, suggesting that many species are vulnerable. In healthy mice, the infections caused only mild, short-lived illness, but in mice lacking key antiviral defenses, all five viruses produced severe disease and death, with one virus proving most harmful. Our tissue studies revealed that each virus damaged organs in different ways, and antibody testing showed strong protection within groups of related viruses but limited protection between groups. Our findings demonstrate that the severity of disease caused by these viruses is linked to how they have evolved and how different their proteins are. Our study provides important tools and knowledge to better understand how these viruses cause disease and support the development of vaccines, treatments, and surveillance strategies to protect against future outbreaks.

## Introduction

Bunyaviruses are a diverse group of arthropod-borne viruses that can cause severe diseases in humans and animals, several of which are considered emerging pathogens of global concern [1,2]. Within this group, the *Bandavirus* genus–classified within the class *Bunyaviricetes*, order *Hareavirales*, and family *Phenuviridae*—has gained attention as a significant global health concern owing to its established or potential pathogenicity in humans [3]. *Bandavirus* is an enveloped [4], negative-sense, single-stranded RNA virus with a genome composed of three segments: small (S), medium (M), and large (L) [5]. According to the most recent classification by the International Committee on Taxonomy of Viruses (2024, https://ictv.global/), nine tick-borne species are currently recognized within this genus: severe fever with thrombocytopenia syndrome virus (SFTSV), Heartland virus (HRTV), Guertu virus (GTV), Hunter Island Group virus (HIGV), Zwiesel bat bandavirus (ZbbV), Bhanja virus (BHAV), Lone Star virus (LSV), Kismaayo virus (KISV), and Razdan virus (RAZV).

*Bandavirus* members exhibit a broad range of epidemiological and disease-related characteristics. SFTSV and HRTV are the most clinically significant viruses and are capable of causing severe febrile illnesses in humans. SFTSV was first isolated in 2009 from patients in China [6,7] and has since been known to be endemic in several countries in East and Southeast Asia [8,9]. In humans, SFTSV infection often leads to acute febrile illness accompanied by severe thrombocytopenia, leukopenia, elevated liver enzyme levels, and multiorgan involvement [10], with case fatality rates of 5–30% [11], particularly among the elderly [12,13]. It is transmitted by *Haemaphysalis longicornis* ticks [14] and infects a wide range of domestic and wild animals [15,16]. HRTV, discovered in the United States in 2009 [17], shows similar clinical features and is transmitted by *Amblyomma americanum* ticks [18]. Although human cases are less common, severe outcomes still occur [19,20]. Other *Bandavirus* members exhibit varying degrees of zoonotic potential. GTV, isolated in China in 2018, can infect mammalian cells and cause disease in mice; serological evidence indicates human exposure, although clinical cases have not been confirmed [21,22]. BHAV, which is distributed across Europe, Asia, and Africa, generally causes mild or subclinical disease in humans and livestock but can occasionally lead to encephalitis [23,24]. LSV, first isolated from *A*. *americanum* in the United States [25,26], has been tentatively linked to encephalitis in an immunocompromised patient [27]. The remaining viruses—HIGV (isolated from *Ixodes eudyptidis* collected from birds in Tasmania, Australia) [28], KISV (from *Rhipicephalus pulchellus* in Somalia) [29], ZbbV (detected in *Eptesicus nilssonii* bat populations in Europe) [30], and RAZV (from *Dermacentor marginatus* in Armenia) [31]—have been identified in diverse hosts and geographical regions across multiple continents, but their zoonotic potential and pathogenicity in humans remain largely uncharacterized.

Animal models are essential for understanding *Bandavirus* infections and evaluating medical countermeasures. A few studies have used immunocompetent models—such as adult C57BL/6 mice [32], adult ferrets [33], and rhesus macaques [34]—which typically develop only mild or transient illness. In contrast, severe or lethal disease has been observed primarily in immunodeficient models, such as IFNAR^−/−^ C57BL/6 mice [35,36] and Stat2^−/−^ hamsters [37]. Aged ferrets have also been developed as a model that closely recapitulates human SFTS [33]. These models have been widely applied to investigate viral pathogenesis and to evaluate the efficacy of vaccines and antiviral agents. Collectively, the complementary use of immunocompetent and immunodeficient systems has provided critical insights into virus–host interactions. However, most research to date has focused on SFTSV and HRTV, limiting our knowledge of the broader genus and underscoring the need for systematic comparative studies to inform the development of broad-spectrum vaccines and therapies.

In this study, we systematically compared five representative *Bandavirus* members–SFTSV, GTV, HRTV, LSV, and BHAV–using both *in vitro* and *in vivo* models to evaluate their infection characteristics and pathogenic differences, including clinical manifestations, viral replication, immune responses, and pathological changes. Our findings offer important insights into the evolutionary patterns and cross-species transmission potential of *Bandavirus* viruses, strengthening our understanding of their epidemiology and informing the development of prevention and control measures. We identified both shared and distinct features of pathogenicity and immunogenicity among these viruses, which could support clinical diagnosis and facilitate development of diagnostic tools. Furthermore, this study establishes the use of animal models to aid research on broad-spectrum antiviral drugs and vaccines. Our study contributes to the advancement of diagnosis, treatment, prevention strategies, and global public health preparedness related to the *Bandavirus* genus.

## Results

### Phylogenetic relationships among *Bandavirus* members

**Figure 1.**
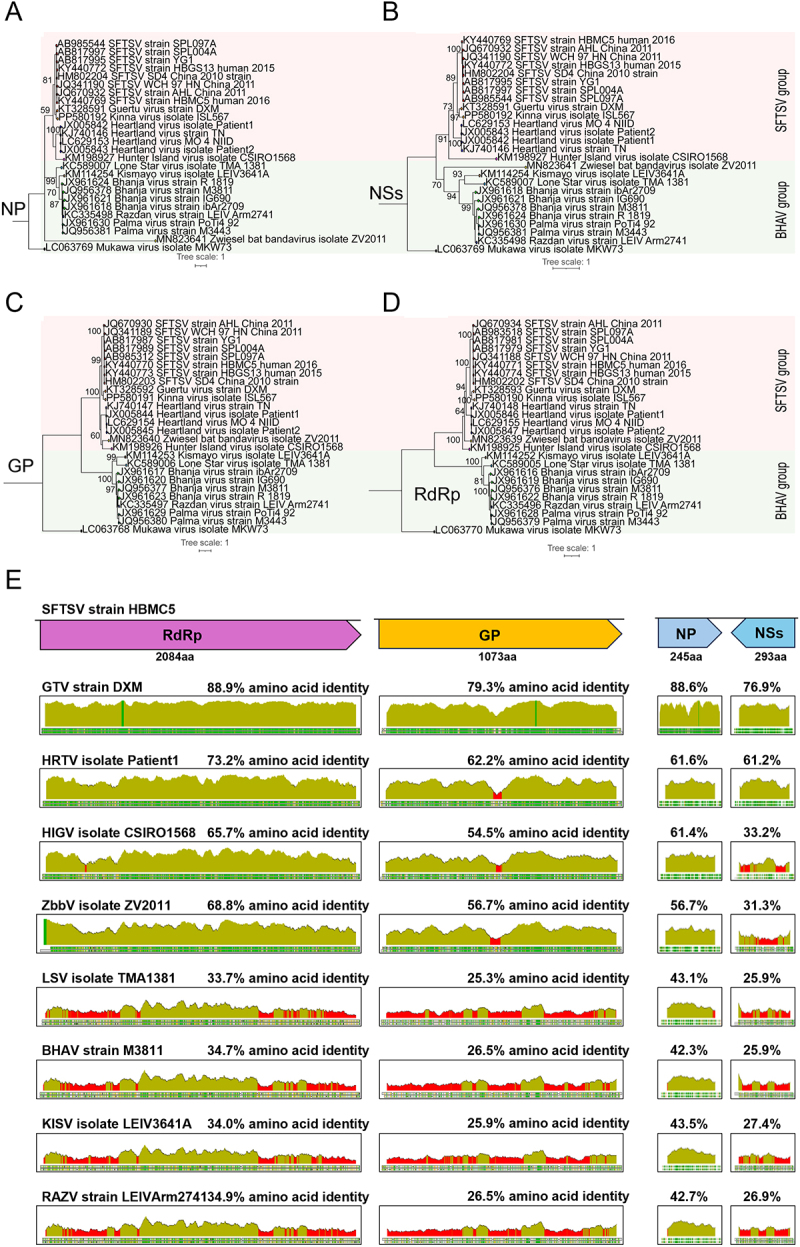
Phylogenetic relationships among *Bandavirus* members based on four viral genes. Maximum likelihood phylogenetic trees were constructed for representative *Bandavirus* members using nucleotide sequences of four viral genes: (A) Nucleoprotein (NP), (B) Nonstructural protein (NSs), (C) glycoprotein (GP), and (D) RNA-dependent RNA polymerase (RdRp). Nucleotide sequences were aligned using MEGA 11.0, phylogenetic trees were generated with PhyloSuite using 1000 bootstrap replicates, and the trees were rooted in Mukawa virus. SFTSV, HRTV, and GTV formed a distinct clade (SFTSV group; pink shading), whereas BHAV, LSV, and related viruses clustered in separate clades (BHAV group; green shading). Scale bars indicate the number of nucleotide substitutions per site. (E) Pairwise amino acid identity and sliding window conservation analysis using SFTSV strain HBMC5 as the reference. Sliding window analysis was performed with a 50-amino-acid sliding window (1-amino-acid step). Green regions indicate high sequence identity (100% identity), greenish-brown regions indicate 30–99% identity, while red regions indicate low sequence identity (below 30% identity). The overall amino acid identity values for each full-length protein are labeled above the corresponding plots.

Phylogenetic analysis of four viral genes—nucleoprotein (NP; Figure 1(A)), non-structural proteins (NSs; Figure 1(B)), glycoprotein (GP; Figure 1(C)), and RNA-dependent RNA polymerase (RdRp; Figure 1(D))—revealed that these viruses could be divided into two groups. SFTSV, GTV, and HRTV consistently clustered together and could be classified as the SFTSV group, whereas BHAV and LSV formed distinct evolutionary branches that were designated as the BHAV group.

To quantify genetic divergence and validate these phylogenetic relationships, we performed pairwise amino acid identity comparisons using SFTSV strain HBMC5 as the reference (Figure 1(E)). Quantitative sequence identity analysis delineated three distinct tiers of conservation: GTV and HRTV exhibited high identity (61.2–88.9%), followed by HIGV and ZbbV (31.3–68.8%), while BHAV, LSV, KISV, and RAZV formed a divergent cluster (25.3–43.5%) (Figure 1(E)). Complementing these findings, pairwise identity heatmap analysis (Fig S1) classified these viruses into two distinct groups (SFTSV group and BHAV group), corroborating the phylogenetic clustering, with this two-group pattern consistently observed across all four viral proteins at both nucleotide and amino acid levels.

### Efficient replication of Bandavirus in human and mammalian cells

**Figure 2.**
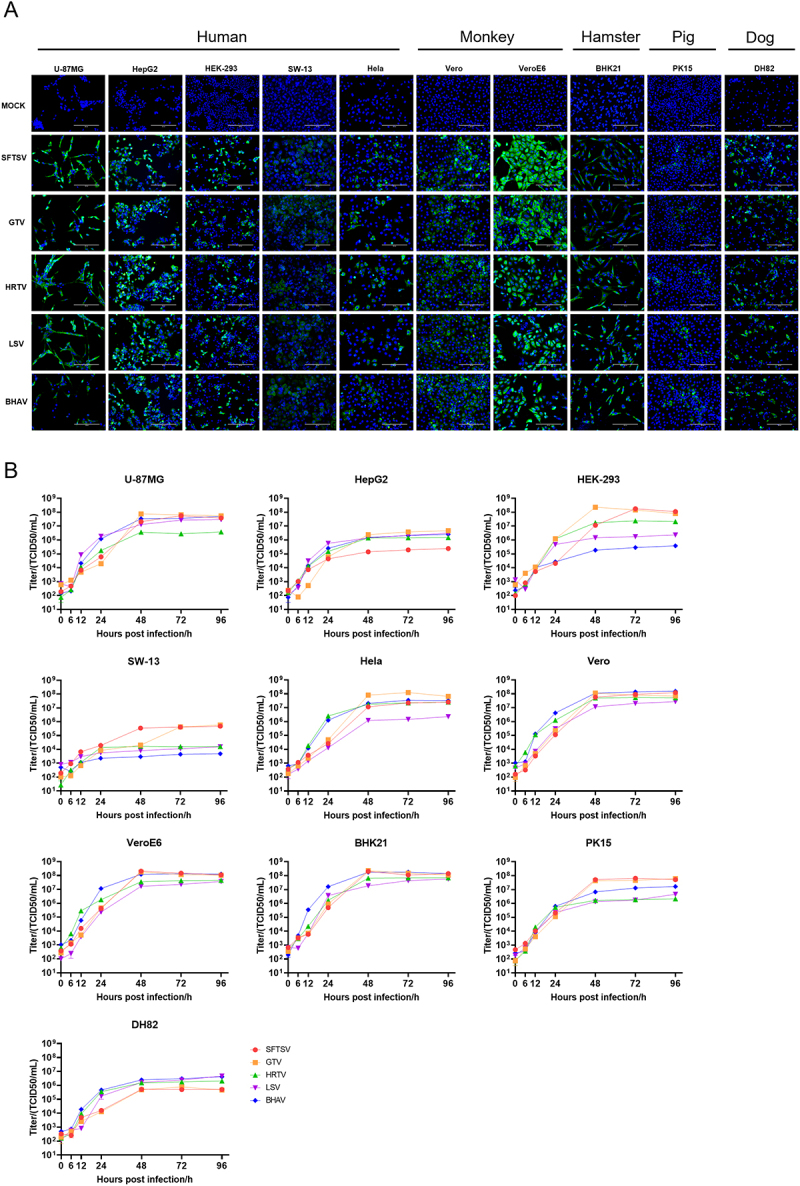
Cell tropism and replication kinetics of five representative *Bandavirus* members. (A) Ten mammalian cell lines—from human (U-87 MG, HepG2, HEK-293, SW13, HeLa), monkey (Vero, VeroE6), hamster (BHK21), pig (PK15), and dog (DH82)—were infected with SFTSV, GTV, HRTV, LSV, or BHAV (multiplicity of infection [MOI] = 5) and fixed at 96 hpi. Cells were stained with a polyclonal antibody against viral NP, followed by an Alexa Fluor-conjugated secondary antibody. Viral NP (green) and nuclear DNA (DAPI; blue) were visualized using fluorescence microscopy. Mock-infected cells served as negative controls. Scale bars: 200 μm. (B) One-step growth curves of the five viruses in selected cell lines. Cells were infected at an MOI of 5, and viral titers in the supernatants were quantified at indicated time points (0–96 hpi) using an endpoint dilution assay (50% tissue culture infectious dose [TCID50]) on Vero cells. Data represent mean ± standard deviation (SD) from three independent experiments. Detailed statistical analysis results for intergroup comparisons are provided in supplementary table S1.

To assess the potential host range and target organs of the *Bandavirus* genus, we evaluated their infectivity and replication efficiency in various host cell types. Successful infection was defined by the detection of viral NP expression via immunofluorescence assay (IFA), as detailed in Materials and Methods. All five viruses could successfully infect human (U87MG, HepG2, HEK-293, SW13, HeLa), monkey (Vero, VeroE6), pig (PK15), dog (DH82), and hamster (BHK21) cells. No antigen signal was detected in uninfected controls, confirming the specificity and reliability of the detection system (Figure 2(A)). No obvious cytopathic effect (CPE) was observed in Vero cells infected with any of the five *Bandavirus* members at MOI 5. The consistency of the virus’s infectivity was determined through IFA (Fig S2). Therefore, Vero cells were selected for the determination of the virus titer. Viral replication kinetics were assessed using one-step growth curves. Across cell lines, all viruses displayed similar replication dynamics, remaining in the amplification phase from 0 to 48 h before transitioning to the plateau phase. Overall, the five *Bandavirus* members replicated efficiently in several cell lines, with the highest viral titers observed in U87MG, HeLa, and PK15 cells, whereas replication in SW13 cells was comparatively limited, suggesting lower permissiveness in this cell type (Figure 2(B) and Table S1). Virus-specific differences were also observed. HRTV exhibited the lowest replication rate in U87MG cells, whereas SFTSV replicated poorly in HepG2 cells. In contrast, SFTSV and GTV consistently achieved higher replication in HEK-293, SW13, and PK15 cells. LSV showed notably lower replication in HeLa cells than the other viruses. Collectively, these findings indicate that although overall replication trends are similar, individual viruses exhibit distinct adaptability across specific host cell types (Figure 2(B) and Table S1).

### Bandavirus exhibits non-lethal infection in C57BL/6 mice and comprises two distinct serotypes

The pathogenicity and antigenic relationships of *Bandavirus* species were investigated using an intraperitoneal infection model in C57BL/6 mice. The pathogenicity and immune responses to SFTSV and GTV in C57BL/6 mice have been characterized in our previous studies, both viruses induced uniformly non-lethal infection with transient viremia and comparable humoral antibody responses [21,38]. Therefore, in this model, we focused on three representative viruses—HRTV, LSV, and BHAV—whose host interactions remain poorly defined. C57BL/6 mice were intraperitoneally infected with 10^7^ TCID_50_ of each virus (the same dose used for SFTSV and GTV in our previous studies [21,38]) and monitored daily for 14 days post-infection.

**Figure 3.**
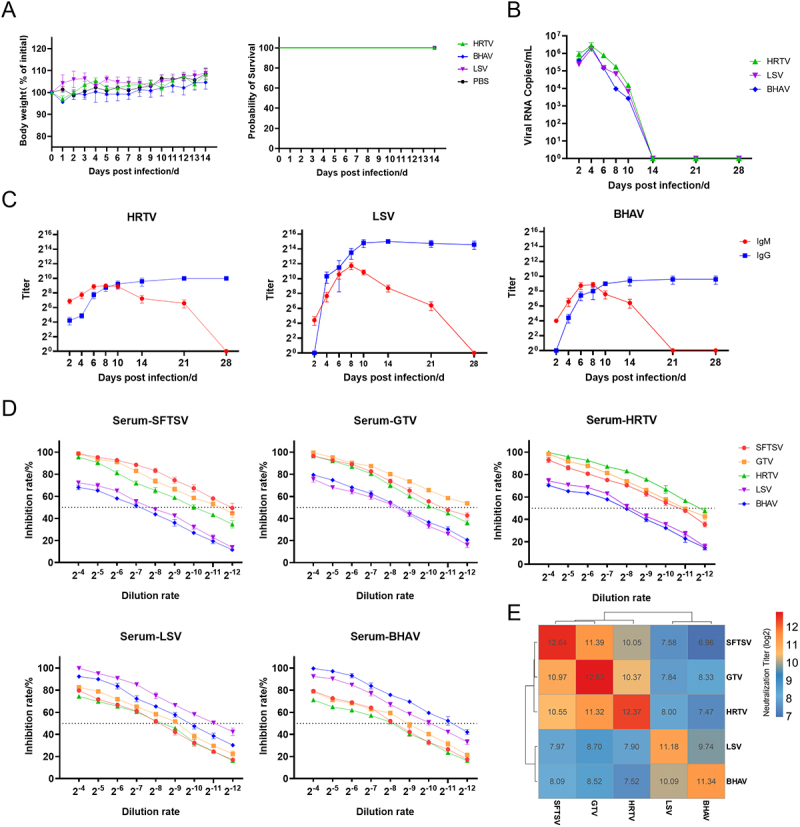
Infection dynamics and humoral immune responses of *Bandavirus* members in C57BL/6 mice. (A) Experimental design and clinical observations. C57BL/6 mice were intraperitoneally infected with 10^7^ TCID_50_ of HRTV, LSV, or BHAV and monitored for 14 d. Body weight was recorded daily. No significant weight loss or overt clinical signs were observed throughout the study. Data are presented as mean ± SD (*n* = 6 per group). (B) Viral RNA copies in serum collected at the indicated time points post-infection were quantified via quantitative real-time PCR (qRT-PCR). Each dot represents the viral RNA level (copies/mL) from an individual mouse. Data are presented as mean ± SD (*n* = 6 per group). (C) Virus-specific IgM and IgG responses. Serum was collected at the indicated time points, and antibody titers were determined via endpoint dilution ELISA. Titers are expressed as log_2_ of the highest dilution with an optical density value at least three times that of the negative control. Symbols represent individual mice (*n* = 6 per group). Data are presented as mean ± SD (*n* = 6 per group). Detailed statistical analysis results for intergroup comparisons are provided in supplementary table S2. (D) Cross-neutralization profiles of *Bandavirus*-specific sera. Sera were collected at 14 dpi from mice infected with SFTSV, GTV, HRTV, LSV, or BHAV and subjected to neutralization assays against each virus. Detailed statistical analysis results for intergroup comparisons are provided in supplementary table S4. (E) Neutralization titers (NT_50_) were defined as the reciprocal of the serum dilution that achieved a 50% reduction in infection. And are expressed as log2-transformed NT50 values. Heatmap visualization reveals two antigenic clusters within the *Bandavirus* genus: one comprising SFTSV, GTV, and HRTV, and the other LSV and BHAV.

No significant clinical signs, including changes in body weight, routine hematological parameters, or biochemical indicators, were observed up to 14 days post-infection (dpi) (Figure 3(A) and Fig S3). All mice survived until euthanasia (Figure 3(A)). Viremia from the three viruses peaked (10^6^-10^7^ copies/mL) on day 4 and was cleared by day 14 (Figure 3(B) and Table S2). IgM antibodies were detected at 2 dpi, peaked between days 6 and 8, and subsequently declined (Figure 3(C) and Fig S4). Notably, HRTV- and LSV-specific IgM antibodies remained undetectable on day 28, whereas BHAV-specific IgM antibodies remained undetectable by day 21. IgG antibodies against HRTV were detected as early as 2 dpi, whereas those against LSV and BHAV appeared on day 4. All IgG antibodies peaked on day 10 and persisted until day 28 (Figure 3(C) and Fig S4). LSV induced significantly higher antibody titers than HRTV or BHAV, with statistically elevated antibody levels observed from day 4 to day 21 post-infection. (Figure 3(C), Fig S4 and Table S3).

To evaluate antibody cross-reactivity, neutralization assays were performed using sera collected at 14 dpi from mice infected with the five *Bandavirus* species. Homologous neutralization elicited robust responses across all groups, with 50% neutralization titers (NT_50_) ranging from 2^−11.18^–2^−12.83^, indicating effective strain-specific immunity (Figure 3(D,E) and Table S4). Cross-neutralization analysis revealed two distinct serological clusters within the genus (Figure 3(E)). Strong reciprocal neutralization was observed among SFTSV, GTV, and HRTV, with sera from any of these viruses effectively neutralizing the others (NT_50_: 2^−10.05^–2^−11.39^). Similarly, moderate mutual cross-neutralization was observed between LSV and BHAV (NT_50_: 2^−9.74^–2^−11.34^). In contrast, cross-neutralization between the two groups was markedly reduced, with NT_50_ values decreasing to 2^−6.96^–2^−8.7^, suggesting limited antigenic overlap (Figure 3(D,E) and Table S4). These findings support the classification of *Bandavirus* members into two serologically distinct subgroups, consistent with their phylogenetic clustering patterns.

### Divergent virulence and hematological profiles of *Bandavirus* in IFNAR^−/−^ C57BL/6 mice

**Figure 4.**
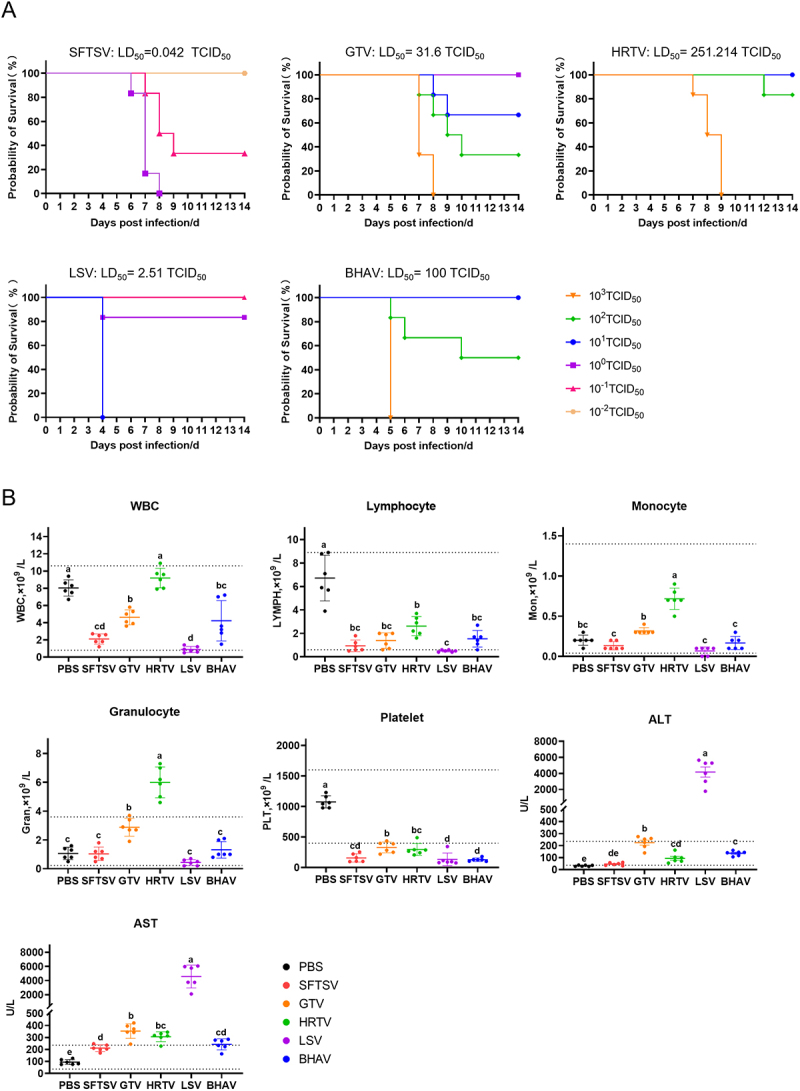
Divergent virulence and hematological profiles of five *Bandavirus* members in IFNAR^−/−^ C57BL/6 mice. (A) Changes in survival rates of mice infected with serial doses of SFTSV, GTV, HRTV, LSV, or BHAV. Mice (*n* = 6 per group) were intraperitoneally injected with increasing doses of virus (10^−2^–10^7^ TCID_50_) and monitored daily for 14 d. Body weights were normalized to 100% on day 0 and are presented as mean ± SD. Survival curves show cumulative mortality across dose groups for each virus. (B) Hematological and biochemical profiles at 3 dpi in mice infected with 1,000 TCID_50_ of each virus. Measured parameters include whole blood cell, lymphocyte, monocyte, granulocyte, and platelet counts, as well as ALT and AST levels. Data are presented as mean ± SD (*n* = 6 per group). Statistical significance was determined using one-way ANOVA followed by Tukey’s multiple comparisons test; groups labeled with different alphabets are significantly different (*p* < 0.05).

To elucidate the pathogenic diversity within the *Bandavirus* genus, we systematically compared the *in vivo* effects of the five viruses in IFNAR^−/−^ C57BL/6 mice. Mice were infected with different doses of each virus, and body weight and survival were recorded daily to calculate the 50% lethal dose (LD_50_) values. The LD_50_ values revealed differences in virulence among the *Bandavirus* species. The virulence ranking based on LD_50_ (in tissue culture infectious dose [TCID50] per mouse) was as follows: SFTSV (0.042 TCID_50_) >LSV (2.51 TCID_50_) >GTV (31.6 TCID_50_) >BHAV (100 TCID50) >HRTV (251.214 TCID_50_) (Figure 4(A) and Fig S5).

To further compare the hematological changes of *Bandavirus* in IFNAR^−/−^ C57BL/6 mice, serum was collected at 3 dpi following infection with the same viral dose. After infection, platelet counts decreased significantly, whereas alanine aminotransferase (ALT) and aspartate aminotransferase (AST) levels markedly increased (Figure 4(B)). LSV infection caused the most pronounced elevation in ALT and AST levels, with average peak values of approximately 4000 U/L (Figure 4(B)). Total white blood cell (WBC), lymphocyte, monocyte, and granulocyte counts in most virus-infected mice remained within the normal range, with the exception of granulocyte counts in the HRTV group, which were significantly elevated (Figure 4(B)). Although no differences were observed in WBC counts between the HRTV and control groups, lymphocyte and WBC counts in the other groups were significantly lower than those in the control group (Figure 4(B)). Granulocyte counts in both the HRTV and GTV groups were significantly higher than those in the control group (Figure 4(B)).

### Tissue tropism and pathological injury profiles of *Bandavirus* in IFNAR^−/−^ C57BL/6 mice

Histopathological examination was performed on tissues collected at 3 dpi, including the liver, spleen, lungs, brain, heart, and kidneys. Across all infections, pathological changes most frequently involved the liver, spleen, lungs, and brain, with varying severity. Common findings included hepatocellular degeneration or vacuolization, inflammatory cell infiltration, disruption of splenic architecture, thickening of alveolar walls, pulmonary hemorrhage or edema, and neuronal degeneration accompanied by perivascular changes. To enable a systematic comparison of lesion severity among different viruses and organs, histopathological changes were semi-quantitatively scored using a four-grade grading system (0–4), as summarized in Table 1 and defined in Supplementary Table S8 [39,40].

**Table 1.** Comprehensive histopathological lesion scores across multiple organs in mice infected with representative *Bandavirus*.

Organ	Lesion type	PBS	SFTSV	GTV	HRTV	LSV	BHAV
Brain	Neuronal shrinkage	0	2	1	1	2	2
	Neuronal edema	0	1	0	0	1	1
	Congestion	0	0	0	0	1	0
	Hemorrhage	0	1	0	1	0	0
Liver	Degeneration	0	1	1	0	3	2
	Necrosis	0	3	2	4	3	3
	Congestion	1	1	1	2	1	3
	Inflammatory cell infiltration	0	1	1	1	1	1
Heart	Degeneration	0	2	1	1	1	1
	Necrosis	0	0	1	1	0	1
	Inflammatory cell infiltration	0	0	1	1	0	0
	Insoluble fibrin deposition	0	0	1	0	0	1
Lung	Inflammatory cell infiltration	0	1	1	1	1	1
	Alveolar wall thickening	0	1	3	2	1	2
	Eosinophilic material	0	1	1	0	1	1
	Congestion	0	1	0	1	0	1
Spleen	Necrosis	0	1	1	1	1	2
	Congestion	0	0	0	0	1	1
	Inflammatory cell infiltration	1	2	1	1	1	1
	White pulp atrophy	0	3	3	3	0	1
Kidney	Edema	0	2	1	1	1	1
	Congestion	0	2	1	1	1	1
	Tubular dilation	0	1	1	1	0	1
	Inflammatory cell infiltration	0	0	1	1	0	1

**Figure 5.**
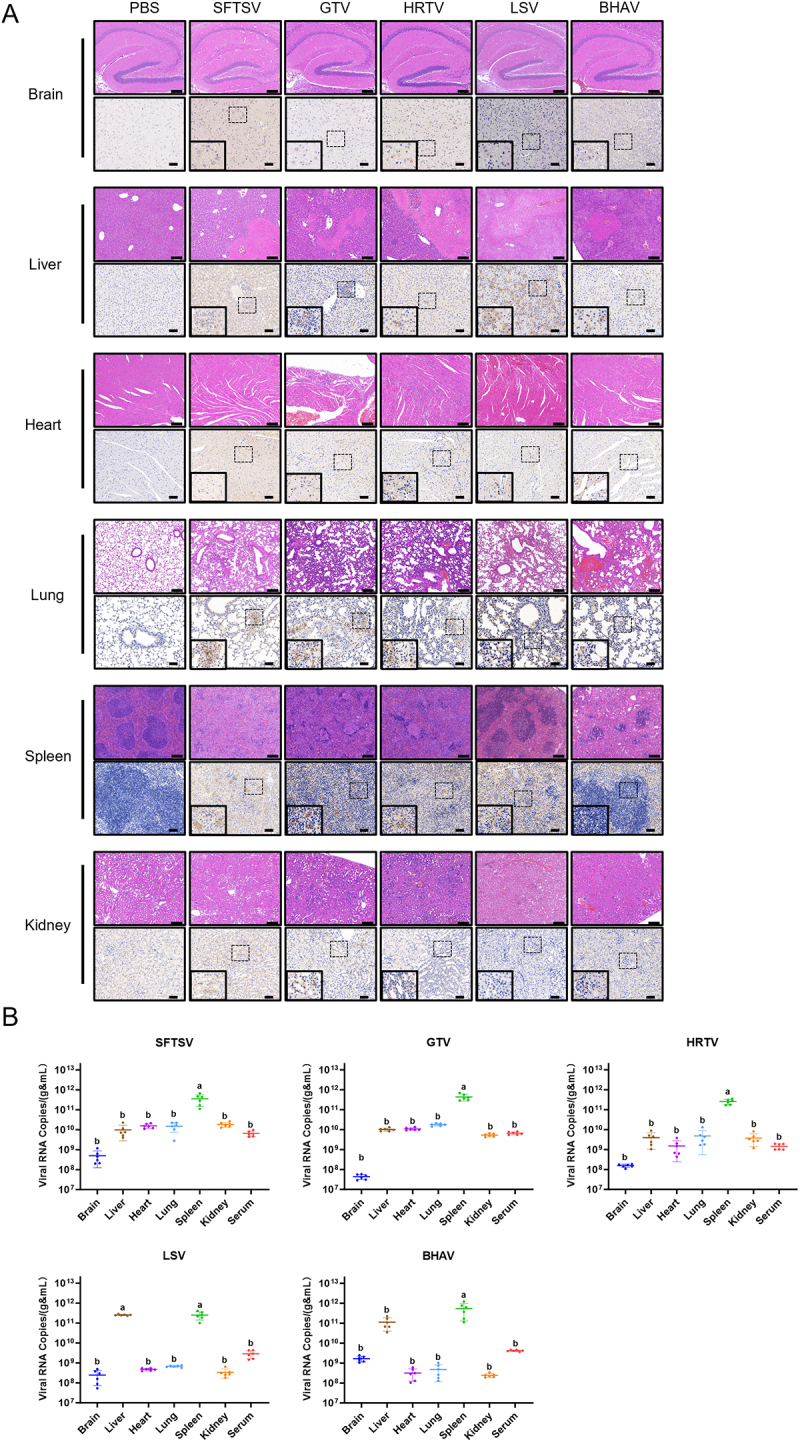
Organ-specific pathology and viral distribution of five *Bandavirus* members in IFNAR^−/−^ C57BL/6 mice. (A) Histopathological lesions and viral antigen distribution in major organs at 3 dpi. Formalin-fixed paraffin-embedded sections were stained with hematoxylin and eosin (H&E; top row) or subjected to immunohistochemistry (IHC; bottom row) using an anti-NP polyclonal antibody. Brown DAB staining indicates NP-positive cells. Insets show higher-magnification views of the boxed regions. Scale bars: 150 μm (H&E), 60 μm (IHC). (B) Tissue viral RNA loads at 3 dpi. Viral RNA was quantified via qRT-PCR in six organs and expressed as log_10_ copies per mg of tissue. Data are represented as mean ± SD (*n* = 6 per group). Statistical significance was determined using one-way ANOVA followed by Tukey’s multiple comparisons test; groups labeled with different alphabets are significantly different (*p* < 0.05).

Despite these shared features, the extent and distribution of lesions varied markedly among the viruses. SFTSV infection caused the most severe and widespread damage, with pronounced lesions in all examined organs, including extensive splenic white pulp depletion, diffuse pulmonary hemorrhage and edema, multifocal neuronal degeneration, severe hepatocellular vacuolization, and myocardial fiber fragmentation. GTV and HRTV shared a pathological profile similar to that of SFTSV, particularly with respect to liver and spleen involvement. In contrast, BHAV induced multisystem pathology with prominent hepatic necrosis, steatosis, and dense inflammatory infiltration, along with pulmonary hemorrhage, splenic disruption, and neuronal shrinkage. LSV infection was characterized by marked hepatic injury–widespread hepatocyte degeneration and necrosis with disruption of hepatic cords–accompanied by cerebral edema and neuronal shrinkage, whereas lesions in the spleen, lungs, and heart were mild to moderate (Figure 5(A)).

High viral loads were detected across all groups, although organ-specific distribution patterns varied among the viruses. In SFTSV-, GTV-, and HRTV-infected mice, the spleen consistently showed the highest titers, whereas the brain exhibited the lowest (Figure 5(B)). LSV- and BHAV-infected mice displayed higher viral loads in the spleen and liver than those in the other organs. Notably, LSV infection resulted in a particularly high viral burden in the liver, consistent with its associated liver pathology (Figure 5(A,B)).

## Discussion

While individual *Bandavirus* members have been extensively characterized, systematic genus-wide comparative analyses remain lacking. Here, we bridge this gap by integrating molecular evolution, *in vitro* tropism, and *in vivo* pathogenicity across five representative viruses—SFTSV, GTV, HRTV, LSV, and BHAV. Our findings provide a comprehensive framework for evaluating the pathogenic characteristics and potential risks of *Bandavirus* infections (Table 2).

**Table 2.** Comparative pathogenic and virological characteristics of *Bandavirus* species.

Parameter	SFTSV	GTV	HRTV	LSV	BHAV
Broad cell line susceptibility	+++	+++	+++	+++	+++
*In vitro* replication efficiency	+++	+++	++	++	++
Weight loss/onset speed in mice	++++	++	++	+++	+++
LD_50_	++++	++	+	++++	+++
Lymphopenia	++	++	+	+++	++
Granulocytes and monocytes elevation	–	++	+++	–	–
Thrombocytopenia	+++	++	++	++++	++++
ALT/AST elevation (liver damage)	+	+++	+++	++++	++
Neurological involvement	++	+	+	++	++
Histopathological damage	++++	+	+	+++	++++
Viral load in major organs	++++	+++	+++	+++	+++
Cross-neutralization reactivity	+++	++++	++	+++	++
Human pathogenicity	++++	+	+++	+	+
Antigenic group	SFTSV Group			BHAV Group	

Each parameter was semiquantitatively scored using “+” symbols to reflect relative intensity across five Bandavirus members (SFTSV, GTV, HRTV, LSV, BHAV): “++++” indicates very high or severe, “+++” high or moderate, “++” mild, “+” minimal, “±” borderline or slight, and “–” absent. Cell line adaptability and in vitro replication efficiency were evaluated based on infection breadth and TCID50 growth curves, respectively. Weight loss and incubation period scores reflected the speed and severity of clinical onset in mice. LD50 scoring was based on the dose required to cause 50% mortality in IFNAR-/- C57BL/6 mice, with lower doses indicating higher virulence. Hematological indicators (leukopenia, thrombocytopenia, and granulocyte and monocyte counts) were scored by the degree of deviation from physiological ranges. Liver enzyme elevation (ALT/AST) indicated hepatocellular damage, while neuroinvasion was assessed by pathological changes or viral presence in the brain. Viral load and tissue damage scores were based on qRT-PCR and histopathological findings, with widespread replication or multi-organ injury assigned higher scores. Cross-neutralization was assessed by 50% neutralization titer (NT50) values, with higher titers reflecting stronger antigenic similarity. Human pathogenicity was scored based on the strength of epidemiological evidence, and antigenic grouping was based on serological cross-reactivity patterns.

Consistent with previous studies on *bandavirus* [15,21,26,29,41], all five viruses replicated efficiently in a broad panel of human- and animal-derived cell lines (Figure 2). While these *in vitro* findings demonstrate widespread cellular susceptibility, they do not fully recapitulate *in vivo* tissue tropism. Nevertheless, the observed broad cellular permissibility highlights the potential for cross-species transmission and underscores the need for enhanced epidemiological surveillance.

Previous studies have established nonlethal and lethal animal models for SFTSV based on host immune status. Immunocompetent animals (mice, rats, hamsters, nonhuman primates) develop mild clinical symptoms upon infection but typically recover fully [34,42,43]. In contrast, lethal outcomes are typically observed in immunodeficient or neonatal mice, such as newborn C57BL/6, IFNAR^− /−^, or Stat2^− /−^ mice, emphasizing the critical role of host immunity in disease severity [35,44]. Studies on HRTV have shown that immunodeficient mice, such as IFNAR^− /−^ mice, develop clinical signs including weight loss, splenomegaly, and thrombocytopenia following infection [36,45]. In contrast, other animals—including raccoons, chickens, rabbits, and hamsters—produce antibodies against HRTV without showing noticeable illness [18,46]. GTV infection in C57BL/6 mice results in detectable viremia accompanied by mild clinical signs [21]. For BHAV, field observations have identified vertebrate hosts including sheep, goats, cattle, African hedgehogs, and ground squirrels, with young ruminants showing febrile and neurological disease; however, no reproducible laboratory model has been established [23,47,48]. In this study, we established two infection models using well-characterized immunocompetent (C57BL/6) and immunodeficient (IFNAR^− /−^ C57BL/6) mouse strains to systematically evaluate the five representative *Bandavirus* members. We observed a consistent pattern: infections were nonlethal with transient viremia and antibody responses in immunocompetent animals but uniformly lethal in IFNAR^− /−^ C57BL/6 mice (Figure 3(A–C) and 4(A) and Table 1). This standardized platform provides a valuable tool for comparative pathogenesis research and vaccine and antiviral evaluation and highlights the risk of severe disease in immunocompromised hosts, emphasizing the need for surveillance and preparedness.

GTV is endemic to Xinjiang, China, near the border with Pakistan, and exhibits serological cross-reactivity with SFTSV [21]. Cross-reactivity among SFTSV, GTV, and HRTV has been demonstrated using monoclonal antibodies targeting conserved motifs [49–51]. In this study, phylogenetic analysis combined with cross-neutralization assays revealed two distinct antigenic groups: the SFTSV group (SFTSV, GTV, HRTV) and the BHAV group (LSV, BHAV) (Figure 1 and 3(D,E) and Table 1). Strong reciprocal neutralization within each group, together with limited intergroup cross-reactivity, suggests the existence of two major antigenic epitopes across the *Bandavirus* genus (Figure 3(D,E)). These findings refine the serological classification of this genus, with immediate practical implications: within-group cross-reactivity challenges diagnostic accuracy, whereas intergroup divergence must be considered for rational vaccine design.

*Bandavirus* species display a clear gradient of virulence in IFNAR^− /−^ C57BL/6 mice, ranging from highly pathogenic (SFTSV, LSV) to intermediate (BHAV) and mild (GTV, HRTV), with disease severity reflected in weight loss, organ pathology, hematological changes, and mortality, but not strictly aligned with phylogeny (Figure 4(A), Fig S5 and Table 1). Importantly, SFTSV-infected IFNAR^−/−^ C57BL/6 mice exhibited severe systemic pathology, characterized by multiorgan failure, marked thrombocytopenia and lymphopenia, as well as elevated liver enzyme levels (ALT and AST) (Figure 4 and 5), closely mirroring the clinical features reported in patients with SFTS [52,53]. The pattern of organ involvement in mice also aligns with previous immunohistochemical findings in fatal human cases [54], indicating that this model closely recapitulates key aspects of human SFTS pathology. While human HRTV infections typically present with leukopenia, thrombocytopenia, lymphopenia, and elevated liver enzyme levels (ALT and AST) [55,56], the same blood changes also occurred in the mouse model (Figure 4 and 5). Notably, we also observed that granulocyte counts (6 × 10^9^/L) were significantly elevated, suggesting that these indicators warrant further investigation. BHAV and LSV infections have been primarily associated with neurotropism and encephalitic outcomes [27,57]. Our findings demonstrate that BHAV and LSV exhibit significant neurotropism in IFNAR^− /−^ C57BL/6 mice, consistent with their reported association with encephalitic outcomes in humans. Importantly, we also observed extensive hepatic injury and systemic viral dissemination, indicating their pathogenicity extends beyond the central nervous system. In contrast, GTV caused only mild disease, yet its serological cross-reactivity with SFTSV raises critical clinical concerns.

Collectively, the IFNAR^−/−^ C57BL/6 mice model reliably recapitulates key features of human *bandavirus* infections and captures interspecies differences in virulence and tissue tropism. While it may overestimate pathogenicity for milder viruses, its standardized performance provides a valuable comparative platform. Notably, the overlapping clinical manifestations and serological cross-reactivity among geographically co-circulating *bandavirus* highlight the urgent need for improved differential diagnostic assays to prevent misdiagnosis and guide appropriate clinical management.

The geographical and host overlaps of these *bandavirus* carry significant public health implications. The co-endemicity of HRTV and LSV with *Amblyomma americanumin* the US [18,26]. SFTSV, GTV, and BHAV show substantial geographical overlap, with shared the shared vectors (*Haemaphysalis/Dermacentor*) and hosts among Asian viruses create ecological niches for co-infection, reassortment, and cross-species transmission [14,21,23]. BHAV’s pan-continental distribution further expands this potential. Coupled with the serological cross-reactivity observed, these overlaps pose risks of misdiagnosis and spillover. Our study provides a critical framework for One Health surveillance to address these threats.

Study limitations include the constrained diversity of animal models, which may not fully represent the spectrum of human disease. Furthermore, sparse clinical data for some emerging viruses limit the validation of model-to-human translatability. Future work should integrate humanized/organoid models and prioritize clinical data collection to refine risk assessments.

In conclusion, this study provides the most extensive comparative analysis to date of the infectivity, immunogenicity, and virulence of *Bandavirus* members. By defining their pathogenic spectra and antigenic relationships, we establish a foundational platform for advancing vaccine development, enhancing diagnostic precision, and informing global surveillance strategies against emerging tick-borne viruses. These findings provide a critical scientific basis for advancing disease prevention and control, optimizing clinical diagnostics, developing therapeutic strategies, and screening drug targets. Importantly, understanding the diverse pathogenic potential and antigenic profiles of these viruses carries direct public health implications, highlighting populations at higher risk, guiding targeted surveillance and early detection programs, and informing the design of effective prevention and intervention strategies to mitigate the impact of future outbreaks.

## Materials and methods

### Cell lines, viruses, and antibodies

Baby hamster kidney (BHK-21, ATCC, CCL-10) and African green monkey kidney (Vero, ATCC, CCL-81) cells were maintained in Dulbecco’s Modified Eagle’s medium (DMEM; Gibco, Grand Island, NY, USA) supplemented with 10% fetal bovine serum (FBS; Gibco, Grand Island, NY, USA). Human embryonic kidney (HEK-293, ATCC, CRL-1573), human cervical adenocarcinoma (HeLa, ATCC, CCL-2), Vero E6 (a clone of Vero, ATCC, CRL-1586), human glioblastoma (U-87 MG, ATCC, HIB-14), human hepatocellular carcinoma (HepG2, ATCC, HB-8065), porcine kidney (PK-15, ATCC, CCL-33), and canine macrophage-like (DH82, ATCC, CRL-10389) cells were all cultured in Eagle’s Minimum Essential medium (Gibco, Grand Island, NY, USA) supplemented with 10% FBS. SW-13 cells (ATCC, CCL-105) were cultured in Leibovitz’s L-15 medium (Gibco, Grand Island, NY, USA) supplemented with 10% FBS. All cell lines were obtained from the American Type Culture Collection (Manassas, VA, USA) and preserved at the National Virus Resource Center (NVRC, China). Mycoplasma contamination was routinely tested using the EZ-PCR Mycoplasma Test Kit (Biological Industries, Kibbutz Beit Haemek, Israel), and all cell lines were confirmed to be mycoplasma-free. Details of all cell lines are summarized in Table S5. All cells were grown at 37°C in a 5% CO_2_ incubator.

The following *Bandavirus* strains were used in this study and preserved at the NVRC. SFTSV strain HBMC5 (CSTR:16533.06.IVCAS06.6311), GTV strain DXM (CSTR:16533.06.IVCAS06.6106), HRTV isolate Patient1 (CSTR:16533.06.IVCAS06.6330), LSV isolate TMA 1381 (CSTR:16533.06.IVCAS06.6335), and BHAV strain M3811 (CSTR:16533.06.IVCAS06.9001) (Table S6). Cell culture experiments involving SFTSV, GTV, and LSV were conducted at a Biosafety Level-2 (BSL-2) laboratory, whereas those with HRTV and BHAV were performed at a Biosafety Level-3 (BSL-3) laboratory. Animal experiments with SFTSV, HRTV, and BHAV were conducted at an Animal Biosafety Level-3 Laboratory (ABSL-3) facility, whereas experiments with GTV and LSV were performed at an Animal Biosafety Level-2 Laboratory (ABSL-2) facility.

Polyclonal primary antibodies against the viral NP of SFTSV, GTV, HRTV, BHAV, and LSV (generated in rabbits or mice) were obtained from the antibody library at NVRC. Secondary antibodies—goat anti-rabbit IgG H&L (Alexa Fluor® 488; ab33503) and goat anti-mouse IgG H&L (Alexa Fluor® 488; ab150113)—were purchased from Abcam (Shanghai, China). Horseradish peroxidase (HRP)-conjugated goat anti-rabbit IgG (H+L) (SA00001) and 4,’6-diamidino-2-phenylindole (DAPI) was obtained from Proteintech (Wuhan, China).

### Phylogenetic analysis

The nucleotide sequences of four proteins in the *Bandavirus* genus—NP, NSs, GP, and RdRp—were selected for phylogenetic analysis. Sequence alignments were performed using MEGA version 11.0. Maximum likelihood phylogenetic trees were constructed using PhyloSuite software, using 1,000 bootstrap replicates to assess branch support and tree reliability. The best-fit substitution models for each protein, determined according to the Bayesian Information Criterion, were as follows: TPM2+G4 for NP, TPM2+R3 for NSs, TIM2+F+G4 for GP, and GTR+F+G4 for RdRp. To quantitatively evaluate the genetic divergence and protein conservation profiles across the *Bandavirus* genus, pairwise amino acid sequence comparisons were performed using SFTSV strain HBMC5 as the reference. Full-length amino acid sequences of RdRp, GP, NP, and NSs from 8 representative *bandavirus* strains were aligned using MAFFT v7 with the FFT-NS-1 algorithm for high accuracy. Sliding window analysis was conducted with a window size of 50 amino acids and a step size of 1 amino acid to visualize the distribution of sequence conservation along each protein. Overall amino acid pairwise similarity was determined by concatenating the 4 bunyavirus protein sequences and running pairwise alignments in Geneious. Pairwise nucleotide and amino acid sequence identities were calculated independently for each of the four viral proteins across 9 representative *bandavirus* strains. Heatmaps were constructed using the pheatmap package in R software, with a color scale ranging from 9% (green) to 100% (red) to indicate increasing sequence identity.

### Cell infection assays and one-step growth curve analysis

To assess the susceptibility of different host cell lines to viral infection, the cells were infected with various viruses at a multiplicity of infection (MOI) of 5. After 2 h of adsorption at 37°C, the inoculum was removed, cells were washed twice with phosphate-buffered saline (PBS) to remove unbound virus, and fresh maintenance medium was added. Mock-infected cells processed under identical conditions served as negative controls for all experiments. For infection efficiency evaluation, cells were fixed with 4% paraformaldehyde at 96 hours post-infection (hpi), and indirect immunofluorescence assay (IFA) was performed. Briefly, cells were permeabilized, blocked, and incubated with a rabbit polyclonal antibody against viral NP, followed by an Alexa Fluor 488-conjugated goat anti-rabbit secondary antibody. Nuclei were counterstained with DAPI (blue). Successful infection was defined as the presence of specific green fluorescence signals corresponding to viral NP expression. Specificity was confirmed by the complete absence of green fluorescence signals in mock-infected negative controls.

To examine the one-step growth curve of the viruses, 50 μL of culture supernatant was collected from each infection group at 0, 6, 12, 24, 48, 72, and 96 hpi. Viral titers were determined using the Reed–Muench method as previously described [58]. All experiments were performed in triplicate.

### Animal experiments

C57BL/6 and IFNAR1^−/−^ C57BL/6 mice were housed under specific pathogen-free (SPF) conditions in the facilities provided and supported by Animal Resource Center of Wuhan Insititute of Virology, Chinese Academy of Sciences. Animal experiments with SFTSV, HRTV, and BHAV were performed in ABSL-3, whereas experiments involving GTV and LSV were conducted in ABSL-2. Female C57BL/6 and IFNAR^−/−^ C57BL/6 mice aged 6–8 weeks (*n* = 6 per group) were used to evaluate the pathogenicity of the five viruses (SFTSV, HRTV, GTV, LSV, and BHAV). Uninfected control mice were administered with the culture medium and kept in a separate cage from the infected mice. C57BL/6 mice were intraperitoneally inoculated with 10^7^ TCID_50_ of each virus, and whole blood was collected at 2, 4, 6, 8, 10, 14, 21, and 28 dpi for antibody, viremia, hematological, biochemical, and pathological analyses. Hematological parameters were assessed using a veterinary automatic hematology analyzer (BC-2800vet; Mindray), and serum biochemical markers were measured using a fully automated biochemical analyzer (Chemray 800; Rayto).

To compare the differences in virulence among the viruses, IFNAR^−/−^ C57BL/6 mice were intraperitoneally infected with a range of viral doses (10^−2^–10^7^ TCID_50_). All infected mice were monitored daily for 14 consecutive days to record changes in body weight and clinical signs, including general appearance, activity level, fur condition, and survival. The LD_50_ of the different viruses in the IFNAR^−/−^ C57BL/6 mice was calculated based on the number of surviving and deceased animals. Based on these LD_50_ values, the mice were intraperitoneally injected with the same dose (10^3^ TCID_50_ per mouse) of each virus to evaluate pathogenic differences. Blood and organ samples were collected at 3 dpi for virological, hematological, and biochemical analyses.

All blood draw and virus inoculation experiments were done under anesthesia using regulated flow of isoflurane: oxygen mix to minimize pain and discomfort to the animals. Following isoflurane (RWD) anesthesia, mice were euthanized by enucleation for exsanguination followed by cervical dislocation. The main experimental procedures were performed by a single operator to ensure consistency in the handling of experimental samples. All animal experiments were carried out in strict accordance with the National Institute of Health guidelines under protocols approved by the Institutional Animal Care and Use Committee of Wuhan Institute of Virology.

### Quantitative real-time PCR (qRT-PCR) analysis

Total RNA was extracted from mouse tissues using RNAiso Plus (Takara, Tokyo, Japan) and from blood samples using the TaKaRa MiniBEST Viral RNA/DNA Extraction Kit (Takara, Tokyo, Japan). qRT-PCR was performed using the One Step TB Green® PrimeScriptTM PLUS RT-PCR Kit (Takara, Tokyo, Japan) with primers targeting the NP gene segment. Viral copy numbers were quantified using an absolute quantification method based on standard curves. Primer sequences are listed in Table S7.

### Hematoxylin and eosin (H&E) and immunohistochemistry (IHC) analysis

H&E staining for histopathological evaluation was performed by ServiceBio (Wuhan, China). Paraffin-embedded tissue sections were deparaffinized, rehydrated, and subjected to antigen retrieval using a citrate buffer (pH 6.0). Endogenous peroxidase activity was quenched with 3% hydrogen peroxide, and nonspecific binding was blocked with 5% bovine serum albumin (BSA). Sections were incubated overnight at 4°C with rabbit antibodies against viral NP as the primary (diluted 1:500), followed by incubation with HRP-conjugated secondary antibodies (diluted 1:500) for 50 min at 25°C. Signal development was performed using the DAB substrate, and nuclei were counterstained with hematoxylin. After dehydration and clearing, slides were mounted and scanned using panoramic MIDI II (3DHISTECH, Budapest, Hungary) to analyze antigen expression.

### Antibody detection using ELISA

Sera collected from infected C57BL/6 mice were used for serological analyses. Vero cells were infected with the different viruses at an MOI of 1 in 96-well plates and cultured for 3 d. After infection, the plates were fixed and stored for antibody detection. Mouse sera were heat-inactivated at 56°C for 30 min. Sera were serially diluted twofold (2°–2^−16^) in PBS containing 1% BSA and 0.05% Tween-20. Antigen-coated plates were equilibrated to room temperature before adding 50 µL of each diluted serum per well. Plates were incubated at 37°C for 2 h, followed by three PBS washes. HRP-conjugated goat anti-mouse IgG (H+L) or IgM (H+L) (Proteintech, Wuhan, China) diluted in PBS with 1% BSA and 0.05% Tween-20 was added and incubated at 37°C for 1.5 h. Plates were washed three times, followed by the addition of 100 µL TMB substrate (Beyotime, Shanghai, China) per well and incubation at room temperature for 20 min. The reaction was stopped with 100 µL of stop solution (Beyotime, Shanghai, China), and absorbance was measured at 450 nm using a microplate reader (Synergy HT; BioTek, Vermont, USA). The endpoint titer was defined as the highest serum dilution yielding an optical density value greater than twice the mean of the negative control. Antibody titers were expressed as the reciprocal of this dilution, and antibody curves were plotted using GraphPad Prism version 8.0.

### Neutralization assay

The cross-neutralizing activity of sera from different *Bandavirus*-infected C57BL/6 mice was evaluated. Mouse sera were serially diluted twofold (2^−4^–2^−12^) in DMEM containing 2% FBS, 100 U/mL penicillin, and 100 µg/mL streptomycin, then mixed at a 1:1 ratio with 100 TCID_50_ of SFTSV, GTV, HRTV, LSV, or BHAV. After 1 h incubation at 37°C, 100 µL of the virus–serum mixtures were added to Vero cells (1 × 10^4^ cells/well) in 96-well plates, with each dilution tested in triplicate. Virus incubated with sera from uninfected mice served as a negative control.

After 72 h of incubation, cells were analyzed via IFA. Images were acquired using the Operetta CLS^TM^ High-Content Analysis System (PerkinElmer, Waltham, MA, USA), and infection rates were calculated as the percentage of NP-positive cells. Neutralization inhibition was determined using the following formula: inhibition (%) = [1 – (infection rate of sample/infection rate of control)] × 100. Neutralization curves and half-maximal inhibitory concentrations were analyzed using GraphPad Prism.

### Statistical analysis

All quantitative data are presented as mean ± standard deviation. Statistical analyses were performed using GraphPad Prism 8.0. Differences between groups were assessed using one-way ANOVA followed by Tukey’s multiple comparisons test. A *p*-value < 0.05 was considered statistically significant.

## Ethics statement

All animal experiments were conducted in accordance with institutional guidelines and were approved in advance by the Ethics Committee of Wuhan Institute of Virology, Chinese Academy of Sciences (Approval numbers: WIVA33202007, WIVA23202303, WIVA33202305, WIVA33202401, WIVAF33202407). Every attempt was made to minimize potential suffering and reduce the number of animals used in the research, following institutional guidelines.

## Supplementary Material

Supplementary_materials.cleans.doc.docx

## Data Availability

The data supporting the findings of this study are available in Science Data Bank at DOI: https://doi.org/10.57760/sciencedb.33710 [59].

## Statement of adherence to arrive guidelines

We have adhered to ARRIVE guidelines. The complete ARRIVE guidelines checklist was included in The ARRIVE Guidelines checklist.
