# Supplementary material for: Divergent virulence and serological profiles reveal pathogenic evolution within the Bandavirus genus
Source: Virulence. 2026 Jul 1;17(1):2696699. doi: 10.1080/21505594.2026.2696699 (PMC13336292; doi:10.1080/21505594.2026.2696699)
Supplement: Supplementary_materials.cleans.doc.docx [file KVIR_A_2696699_SM2197.docx]

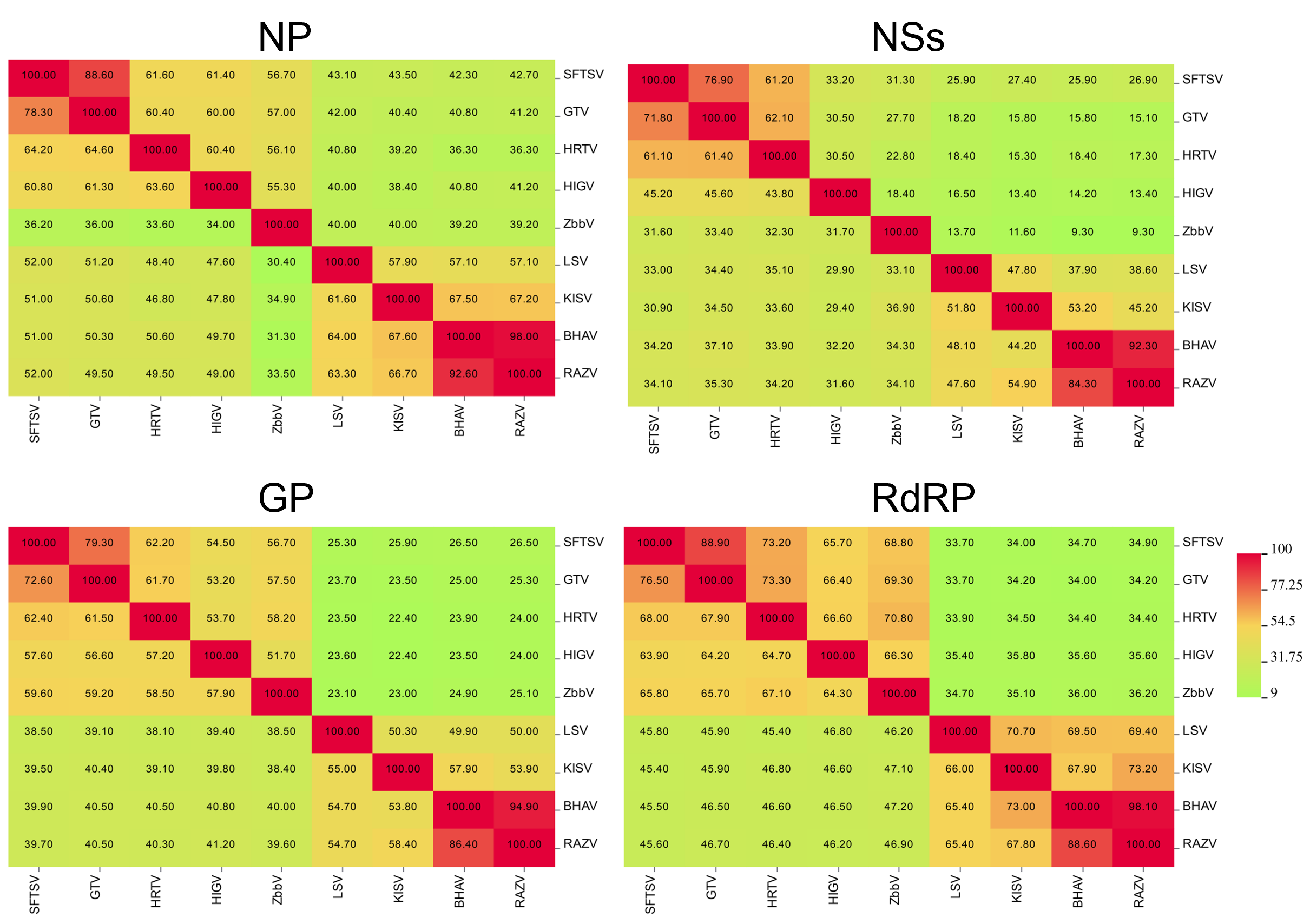


Figure S1. Nucleotide and amino acid sequences of NP, NSs, GP and RdRP from nine representative *Bandavirus* strains were aligned separately, and calculated pairwise sequence identities. Each heatmap corresponds to one viral protein, with the left half displaying nucleotide sequence identities and the right half displaying amino acid sequence identities. The color gradient from green to red indicates increasing sequence identity ranging from 9% to 100%. All four heatmaps consistently delineate two distinct genotypic clusters: the SFTSV-related cluster (SFTSV, GTV, HRTV, HIGV, ZbbV) and the BHAV-related cluster (LSV, BHAV, KISV, RAZV).


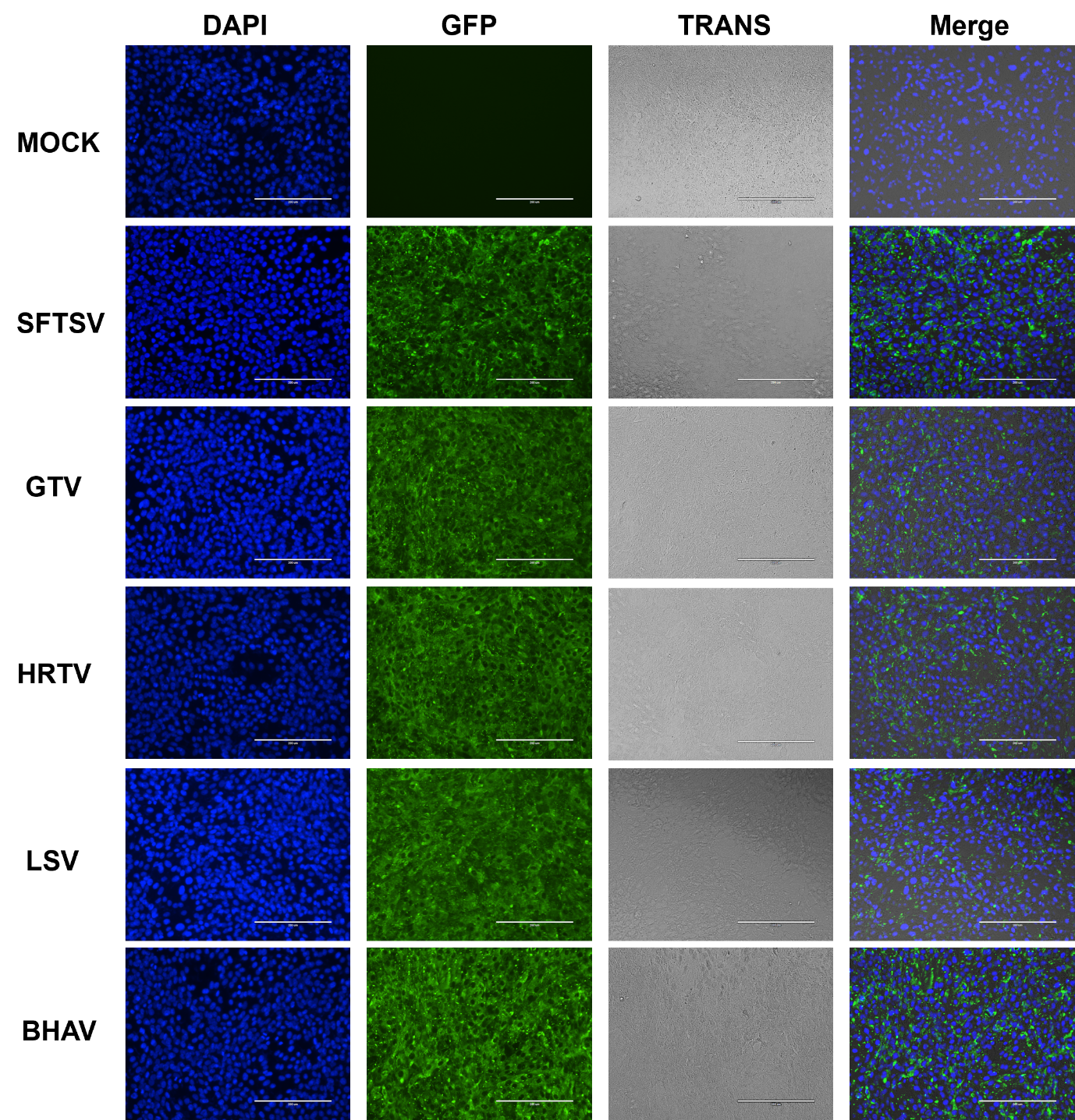


Figure S2. Vero cells were infected with each *Bandavirus* strain at MOI 5 and fixed at the 96 hpi. Panels show corresponding DAPI nuclear staining, viral NP immunofluorescence (green), bright-field view, and merged images for each virus, with uninfected Vero cells as the negative control. No apparent cytopathic morphological changes were observed in infected Vero cells, while strong NP fluorescence indicated efficient viral infection. The intact cell morphology and consistent infection efficiency confirm equivalent susceptibility of Vero cells to all five viruses, supporting the reliability and comparability of subsequent TCID_50_ assays.


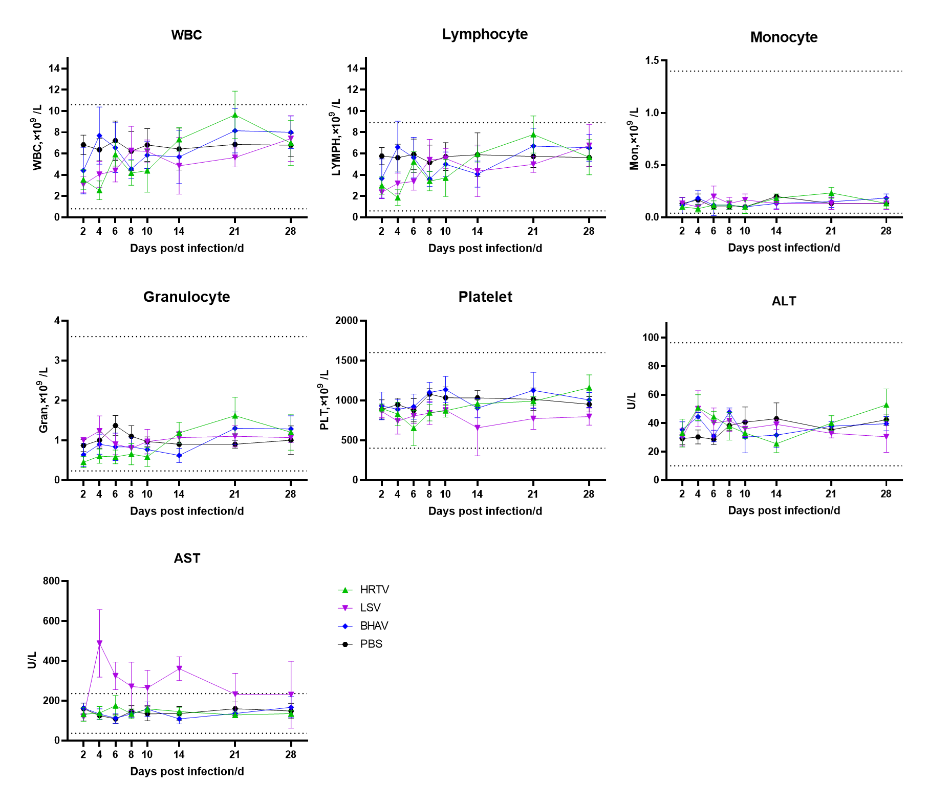


Figure S3. Hematological and biochemical profiles of C57BL/6 mice infected with HRTV, LSV, or BHAV. C57BL/6 mice were intraperitoneally infected with 10^7^ TCID_50_ of HRTV, LSV, or BHAV, and PBS-treated mice served as negative controls. Peripheral blood was collected at the indicated time points post infection for hematological and biochemical analyses. Parameters measured included white blood cell (WBC) count, lymphocytes (absolute number and percentage), monocytes (absolute number and percentage), granulocytes (absolute number and percentage), red blood cells (RBC), platelets (PLT), and liver enzymes (ALT, AST). Data are presented as mean ± SD (n = 6 per group). Statistical significance was determined using one-way ANOVA followed by multiple comparisons.


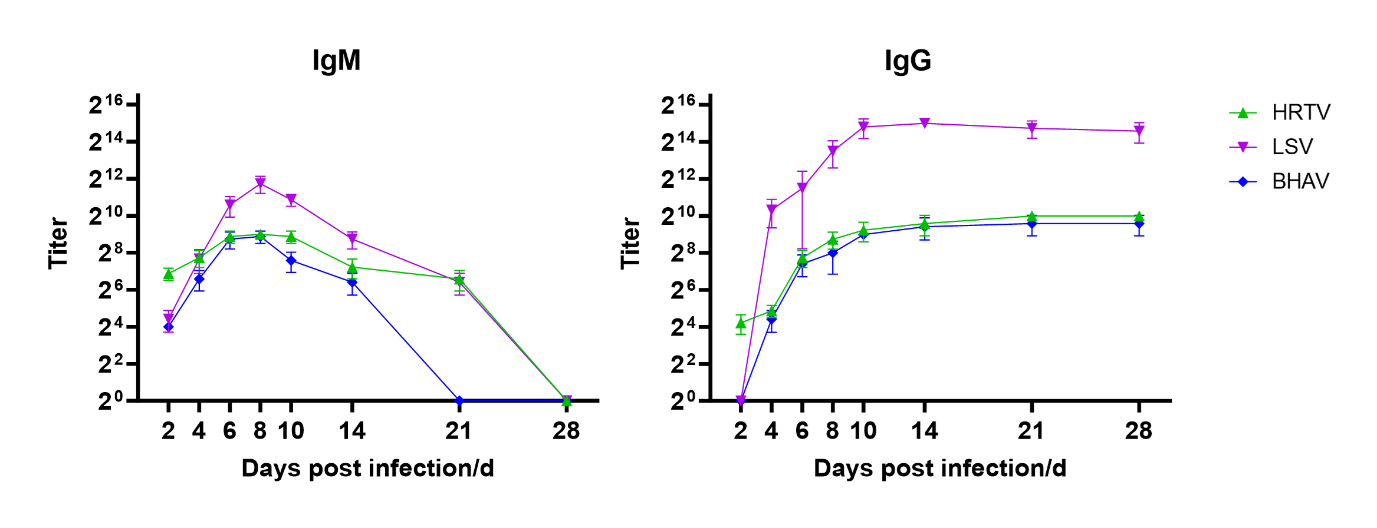


Figure S4. Virus-specific IgM and IgG responses. Serum was collected at the indicated time points, and antibody titers were determined via endpoint dilution ELISA. Titers are expressed as log_2_ of the highest dilution with an optical density value at least three times that of the negative control. Symbols represent individual mice (n = 6 per group). Data are presented as mean ± SD (n = 6 per group). Detailed statistical analysis results for intergroup comparisons are provided in Supplementary Table 3.


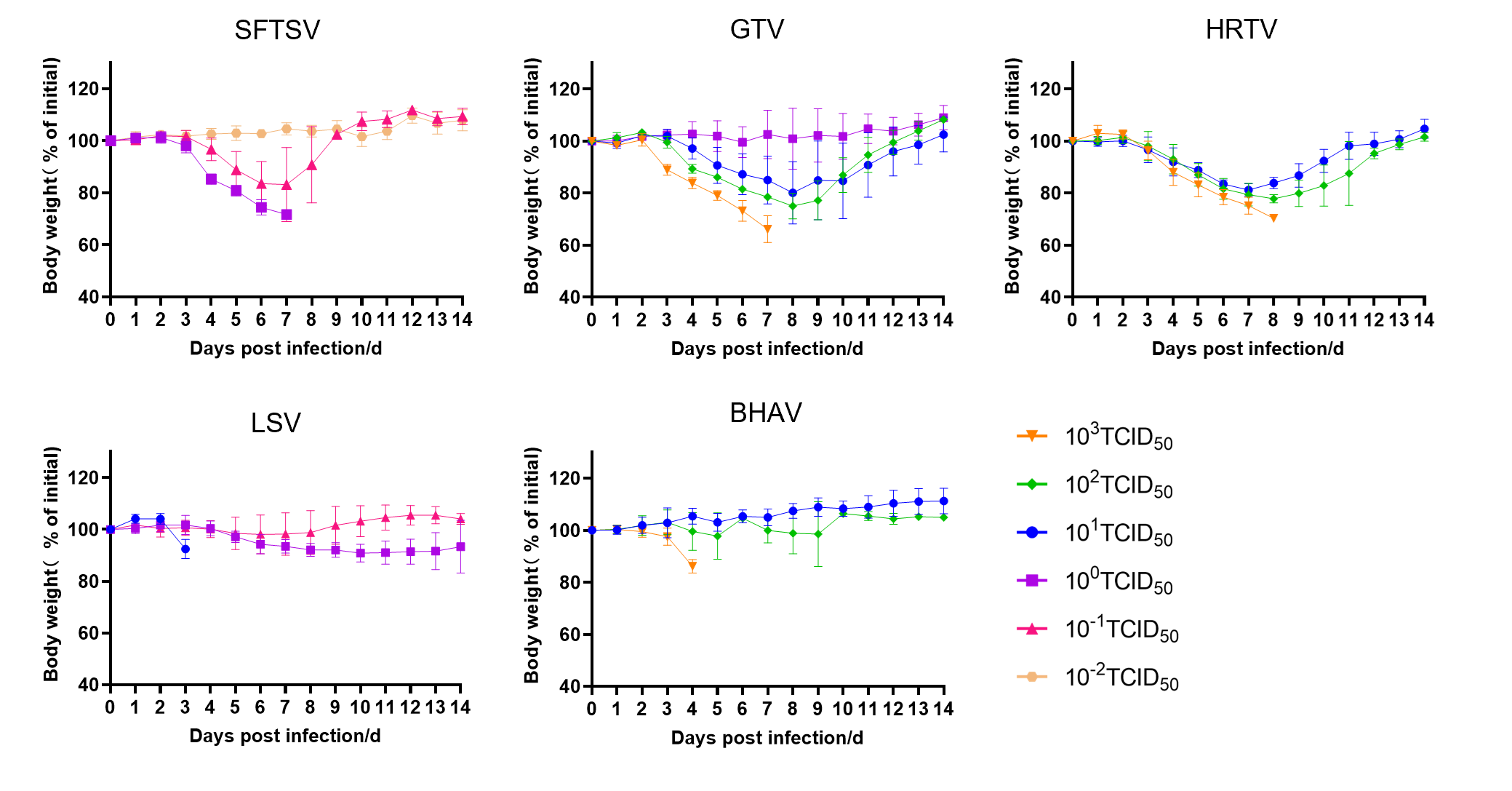


Figure S5. Female IFNAR⁻^/^⁻ C57BL/6 mice aged 6–8 weeks (n = 6 per group) were intraperitoneally infected with serial 10-fold dilutions of SFTSV, GTV, HRTV, LSV, or BHAV ranging from 10^-2^ to 10^7^ TCID_50_ per mouse. Mice were monitored daily for 14 days post-infection, and body weight changes were recorded. Body weights were normalized to 100% of the initial weight on day 0 and are presented as mean ± standard deviation (SD). This dose-gradient experiment was performed to calculate the 50% lethal dose (LD_50_) for each virus, enabling comparison of virulence among the five *Banda*virus species. Survival curves corresponding to these infections are presented in Figure 4A.

Table S1 Statistical analysis of viral titer differences (Fig 2B)

|  | **U-87MG** | | | | |  | **HepG2** | | | | |  | **HEK-293** | | | | |  | **SW-13** | | | | |  | **Hela** | | | | |
| --- | --- | --- | --- | --- | --- | --- | --- | --- | --- | --- | --- | --- | --- | --- | --- | --- | --- | --- | --- | --- | --- | --- | --- | --- | --- | --- | --- | --- | --- |
| **hpi** | **SFTSV** | **GTV** | **HRTV** | **LSV** | **BHAV** |  | **SFTSV** | **GTV** | **HRTV** | **LSV** | **BHAV** |  | **SFTSV** | **GTV** | **HRTV** | **LSV** | **BHAV** |  | **SFTSV** | **GTV** | **HRTV** | **LSV** | **BHAV** |  | **SFTSV** | **GTV** | **HRTV** | **LSV** | **BHAV** |
| **0** | **b** | **a** | **b** | **a** | **b** |  | **a** | **a** | **a** | **a** | **a** |  | **b** | **ab** | **b** | **a** | **b** |  | **bc** | **c** | **c** | **a** | **ab** |  | **a** | **a** | **a** | **a** | **a** |
| **6** | **a** | **a** | **b** | **a** | **b** |  | **ab** | **c** | **a** | **c** | **bc** |  | **b** | **a** | **b** | **b** | **b** |  | **a** | **b** | **b** | **a** | **b** |  | **a** | **bc** | **ab** | **c** | **ab** |
| **12** | **b** | **b** | **b** | **a** | **b** |  | **bc** | **c** | **b** | **a** | **b** |  | **b** | **a** | **ab** | **ab** | **a** |  | **a** | **c** | **c** | **b** | **c** |  | **c** | **c** | **a** | **c** | **b** |
| **24** | **c** | **c** | **c** | **a** | **b** |  | **b** | **b** | **b** | **a** | **b** |  | **c** | **a** | **a** | **b** | **c** |  | **a** | **bc** | **ab** | **c** | **c** |  | **c** | **c** | **a** | **c** | **b** |
| **48** | **b** | **a** | **b** | **b** | **b** |  | **c** | **a** | **b** | **b** | **b** |  | **b** | **a** | **b** | **b** | **b** |  | **a** | **b** | **b** | **b** | **b** |  | **b** | **a** | **b** | **b** | **b** |
| **72** | **a** | **a** | **c** | **b** | **b** |  | **c** | **a** | **b** | **b** | **b** |  | **a** | **a** | **b** | **b** | **b** |  | **a** | **a** | **b** | **b** | **b** |  | **bc** | **a** | **bc** | **c** | **b** |
| **96** | **b** | **a** | **c** | **b** | **a** |  | **d** | **a** | **cd** | **b** | **bc** |  | **a** | **b** | **c** | **c** | **c** |  | **a** | **a** | **b** | **b** | **b** |  | **b** | **a** | **b** | **c** | **b** |
|  | **Vero** | | | | |  | **VeroE6** | | | | |  | **BHK21** | | | | |  | **PK15** | | | | |  | **DH82** | | | | |
| **hpi** | **SFTSV** | **GTV** | **HRTV** | **LSV** | **BHAV** |  | **SFTSV** | **GTV** | **HRTV** | **LSV** | **BHAV** |  | **SFTSV** | **GTV** | **HRTV** | **LSV** | **BHAV** |  | **SFTSV** | **GTV** | **HRTV** | **LSV** | **BHAV** |  | **SFTSV** | **GTV** | **HRTV** | **LSV** | **BHAV** |
| **0** | **c** | **c** | **ab** | **bc** | **a** |  | **b** | **b** | **ab** | **b** | **a** |  | **a** | **b** | **ab** | **a** | **b** |  | **a** | **b** | **b** | **ab** | **ab** |  | **a** | **a** | **a** | **a** | **a** |
| **6** | **b** | **b** | **a** | **b** | **b** |  | **bc** | **bc** | **a** | **c** | **b** |  | **a** | **a** | **a** | **b** | **a** |  | **a** | **a** | **a** | **a** | **a** |  | **b** | **ab** | **b** | **ab** | **a** |
| **12** | **c** | **c** | **a** | **bc** | **a** |  | **b** | **b** | **a** | **b** | **b** |  | **b** | **b** | **b** | **b** | **a** |  | **b** | **b** | **a** | **b** | **b** |  | **bc** | **bc** | **b** | **c** | **a** |
| **24** | **c** | **c** | **a** | **bc** | **a** |  | **b** | **b** | **b** | **b** | **a** |  | **b** | **b** | **b** | **b** | **a** |  | **c** | **c** | **ab** | **bc** | **a** |  | **c** | **c** | **ab** | **bc** | **a** |
| **48** | **b** | **a** | **b** | **c** | **a** |  | **a** | **a** | **b** | **c** | **ab** |  | **a** | **a** | **b** | **b** | **a** |  | **a** | **a** | **b** | **b** | **b** |  | **c** | **c** | **b** | **b** | **a** |
| **72** | **b** | **bc** | **c** | **d** | **a** |  | **a** | **a** | **b** | **b** | **a** |  | **abc** | **ab** | **bc** | **c** | **a** |  | **a** | **b** | **c** | **c** | **c** |  | **c** | **c** | **b** | **ab** | **a** |
| **96** | **a** | **b** | **bc** | **c** | **a** |  | **a** | **a** | **b** | **b** | **a** |  | **a** | **ab** | **bc** | **c** | **a** |  | **a** | **a** | **c** | **bc** | **b** |  | **c** | **c** | **b** | **a** | **a** |

Statistical significance was determined using one-way ANOVA followed by Tukey’s multiple comparisons test; groups labeled with different alphabets are significantly different (*p* < 0.05).

Table S2 Statistical analysis of Viral RNA copies differences (Fig 3B)

| **Viral RNA copies** | | | |
| --- | --- | --- | --- |
| **dpi** | **HRTV** | **LSV** | **BHAV** |
| **2** | **a** | **b** | **b** |
| **4** | **a** | **a** | **a** |
| **6** | **a** | **b** | **b** |
| **8** | **a** | **b** | **c** |
| **10** | **a** | **b** | **c** |
| **14** | **\** | **\** | **\** |
| **21** | **\** | **\** | **\** |
| **28** | **\** | **\** | **\** |

Statistical significance was determined using one-way ANOVA followed by Tukey’s multiple comparisons test; groups labeled with different alphabets are significantly different (*p* < 0.05).

Table S3 Statistical analysis of IgM and IgG differences (Fig S2)

|  | **IgM** | | |  | **IgG** | | |
| --- | --- | --- | --- | --- | --- | --- | --- |
| **dpi** | **HRTV** | **LSV** | **BHAV** |  | **HRTV** | **LSV** | **BHAV** |
| **2** | **a** | **b** | **b** |  | **a** | **b** | **b** |
| **4** | **a** | **a** | **b** |  | **b** | **a** | **b** |
| **6** | **b** | **a** | **b** |  | **b** | **a** | **b** |
| **8** | **b** | **a** | **b** |  | **b** | **a** | **b** |
| **10** | **b** | **a** | **b** |  | **b** | **a** | **b** |
| **14** | **b** | **a** | **b** |  | **b** | **a** | **b** |
| **21** | **a** | **a** | **b** |  | **b** | **a** | **b** |
| **28** | **\** | **\** | **\** |  | **b** | **a** | **b** |

Statistical significance was determined using one-way ANOVA followed by Tukey’s multiple comparisons test; groups labeled with different alphabets are significantly different (*p* < 0.05).

Table S4 Statistical analysis of Cross-neutralization differences (Fig 3D)

|  | **Serum-SFTSV** | | | | |  | **Serum-GTV** | | | | |  | **Serum-HRTV** | | | | |  | **Serum-LSV** | | | | |  | **Serum-BHAV** | | | | |
| --- | --- | --- | --- | --- | --- | --- | --- | --- | --- | --- | --- | --- | --- | --- | --- | --- | --- | --- | --- | --- | --- | --- | --- | --- | --- | --- | --- | --- | --- |
| **dilution** | **SFTSV** | **GTV** | **HRTV** | **LSV** | **BHAV** |  | **SFTSV** | **GTV** | **HRTV** | **LSV** | **BHAV** |  | **SFTSV** | **GTV** | **HRTV** | **LSV** | **BHAV** |  | **SFTSV** | **GTV** | **HRTV** | **LSV** | **BHAV** |  | **SFTSV** | **GTV** | **HRTV** | **LSV** | **BHAV** |
| **2^-4^** | **a** | **a** | **a** | **b** | **b** |  | **a** | **a** | **a** | **b** | **b** |  | **b** | **a** | **a** | **c** | **c** |  | **c** | **c** | **d** | **a** | **b** |  | **c** | **c** | **d** | **b** | **a** |
| **2^-5^** | **a** | **ab** | **b** | **c** | **c** |  | **ab** | **a** | **b** | **c** | **d** |  | **c** | **b** | **a** | **d** | **e** |  | **d** | **c** | **d** | **a** | **b** |  | **c** | **c** | **d** | **b** | **a** |
| **2^-6^** | **a** | **a** | **b** | **c** | **c** |  | **a** | **a** | **a** | **b** | **b** |  | **c** | **b** | **a** | **d** | **e** |  | **d** | **c** | **d** | **a** | **b** |  | **c** | **c** | **d** | **b** | **a** |
| **2^-7^** | **a** | **b** | **c** | **d** | **d** |  | **ab** | **a** | **b** | **c** | **c** |  | **c** | **b** | **a** | **d** | **e** |  | **cd** | **c** | **d** | **a** | **b** |  | **c** | **c** | **d** | **b** | **a** |
| **2^-8^** | **a** | **b** | **c** | **d** | **d** |  | **b** | **a** | **b** | **c** | **c** |  | **b** | **b** | **a** | **c** | **c** |  | **d** | **c** | **d** | **a** | **b** |  | **d** | **c** | **d** | **b** | **a** |
| **2^-9^** | **a** | **b** | **c** | **d** | **e** |  | **b** | **a** | **b** | **c** | **c** |  | **b** | **b** | **a** | **c** | **c** |  | **d** | **c** | **d** | **a** | **b** |  | **d** | **c** | **d** | **b** | **a** |
| **2^-10^** | **a** | **b** | **c** | **d** | **d** |  | **b** | **a** | **c** | **d** | **d** |  | **b** | **b** | **a** | **c** | **c** |  | **c** | **c** | **c** | **a** | **b** |  | **d** | **c** | **d** | **b** | **a** |
| **2^-11^** | **a** | **a** | **b** | **c** | **c** |  | **b** | **a** | **b** | **c** | **c** |  | **b** | **b** | **a** | **c** | **c** |  | **d** | **c** | **d** | **a** | **b** |  | **cd** | **c** | **d** | **b** | **a** |

Statistical significance was determined using one-way ANOVA followed by Tukey’s multiple comparisons test; groups labeled with different alphabets are significantly different (*p* < 0.05).

Table S5 Number of cell lines utilized in the experiment

| Cell name | biological origin | Organizational and biological origin | ATCC No. | NVRC No. |
| --- | --- | --- | --- | --- |
| U-87MG | Human | Glioblastoma-Astrocytoma | HIB-14 | 16533. 09. IVCAS9. 0123 |
| HepG2 |  | Hepatocellular Carcinoma | HB-8065 | 16533. 09. IVCAS9. 0092 |
| HEK-293 |  | Embryonic Kidney | CRL-1573 | 16533. 09. IVCAS9. 0093 |
| SW-13 |  | Kidney | CCL-105 | 16533. 09. IVCAS9. 0144 |
| HeLa |  | Cervical Adenocarcinoma | CCL-2 | 16533. 09. IVCAS9. 0090 |
| Vero | African Green Monkey | Kidney | CCL-81 | 16533. 09. IVCAS9. 0134 |
| Vero E6 |  | Kidney | CRL-1586 | 16533. 09. IVCAS9. 0130 |
| DH82 | Dog | Malignant Histiocytosis | CRL-10389 | 16533. 09. IVCAS9. 0084 |
| BHK-21 | Hamster | Kidney | CCL-10 | 16533. 09. IVCAS9. 0088 |
| PK15 | Pig | Kidney | CCL-33 | 16533. 09. IVCAS9.0180 |

Table S6 Number of virus utilized in the experiment

| Virus | Strain | GenBank | NVRC No. |
| --- | --- | --- | --- |
| SFTSV | HBMC5 | S：KY440769；M： KY440770；  L：KY440771 | 16533.06.IVCAS06.6311 |
| GTV | DXM | S：KT328591；M：KT328592；  L：KT328593 | 16533.06.IVCAS06.6106 |
| HRTV | Patient1 | S：JX005842；M：JX005844；  L：JX005846 | 16533.06.IVCAS06.6330 |
| LSV | TMA 1381 | S：KC589007；M：KC589006；  L：KC589005 | 16533.06.IVCAS06.6335 |
| BHAV | M3811 | S：JQ956378；M：JQ956377；  L：JQ956376 | 16533.06.IVCAS06.9001 |

Table S7 A list of conventional PCR primers used in this study.

| Primer name | Sequence(5’-3’) |
| --- | --- |
| SFTSV-S-qF | CTGGGCAATGGAAACCGGAAG |
| SFTSV-S-qR | CAATGAGGAAGAAGTGAACAAGT |
| GTV-S-qF | ACTCTGAGCCACCCTGAC |
| GTV-S-qR | TGTATTTGCCCTCACTCG |
| HRTV-S-qF | CCTGTTGGGCCAGCAATCAT |
| HRTV-S-qR | GTGGCATTTGGGAAGAAGAGG |
| LSV-S-qF | TATCACCAAAGCCTTCTTTG |
| LSV-S-qR | GCTGGCAGAGTTCATGGCAG |
| BHAV-S-qF | ATGATGAAGTTGTTGGCGGATTGG |
| BHAV-S-qR | CTTTCCAGCCATCCTTGTCAATGTC |

Table S8 Semi-quantitative histopathological grading system.

| **Grade (Score)** | **Category** | **Description** |
| --- | --- | --- |
| **0** | Within normal limits | Under the experimental conditions, considering factors such as the age, sex, and strain of the animals, the tissue is regarded as normal. Changes that may be considered abnormal under other conditions are interpreted as within normal limits in this context. |
| **1** | Minimal | Changes are present but only slightly exceed the normal range. |
| **2** | Mild | Lesions are observable but not severe. |
| **3** | Moderate | Lesions are clearly evident and are likely to progress to more severe changes. |
| **4** | Severe | Lesions are extremely severe, with pathological changes involving the majority or the entire tissue or organ. |

Reference：Mann PC, et al. International Harmonization of Nomenclature and Diagnostic Criteria for Lesions in Rats and Mice (INHAND). Chinese translation edited by Yang L, Zhou X, Zhao D. Beijing: China Agriculture Press; 2019.
